# Real-Time Scene Monitoring for Deaf-Blind People

**DOI:** 10.3390/s22197136

**Published:** 2022-09-21

**Authors:** Khaled Kassem, Piergiorgio Caramazza, Kevin J. Mitchell, Mitch Miller, Azadeh Emadi, Daniele Faccio

**Affiliations:** 1 School of Physics and Astronomy, University of Glasgow, Glasgow G12 8QQ, UK; 2 School of Culture and Creative Arts, University of Glasgow, Glasgow G12 8QQ, UK

**Keywords:** mmWave, radar, sensory impairment, haptic feedback, guidance, deafblind, wearable

## Abstract

It is estimated that at least 15 million people worldwide live with severe deaf-blindness, with many more experiencing varying degrees of deaf-blindness. The existing options of assistance are mostly limited to walking canes, guide dogs and human care. We propose a wearable device which harnesses a multi-antenna mmWave radar transceiver and a haptic feedback array for real time detection of a person moving within a scene. We present our findings from a series of workshops with participants classed with multi-sensory impairments (MSI), to demonstrate the relative success of this approach and its potential for integration into existing assistance for the MSI of the future.

## 1. Introduction

Multi-sensory impairment (MSI), also commonly known as deafblindness, describes various degrees of vision and hearing impairments that can complicate everyday life [1]. Estimates suggest as many as 15 million people worldwide manifest severe deaf-blindness, with up to 450,000 estimated in the UK alone (Operational Research Society (2017)); many more are affected by varying degrees of the condition [2,3]. A careful application of novel technologies can make it possible to give those living with MSI a greater sense of autonomy and safety, and so greatly increases their quality of life [4]. Varying strengths of vision aids such as glasses, magnifiers and text-to-speech technologies are, whilst ubiquitous, often inadequate for people with multi-sensory impairment. Historically speaking, the most rudimentary but effective aid to help those with MSI navigate through their lives is the white cane. This of course provides minimal information on one’s surroundings, with no real ability to sense dynamic objects such as people in a room, or in fact detect anything above the waist.

During our meetings with people that have MSI, we ascertained that there exists the opportunity to confront these challenges head on: by applying novel cost effective and portable technologies and algorithms to develop an aid which can not only detect the presence of obstacles and people in a scene, but clearly communicate this information to people with MSI. Such a pursuit has the potential to revolutionise how these people can interact with their environment, and unlock access to sensory information that would otherwise remain out of reach.

In this work, we propose a scene monitoring device which utilises the people-tracking capabilities of an mmWave band radar transceiver and an array of haptic feedback motors, to detect moving subjects in a scene and relay this information through a wearable hat. We begin by analysing the state of the art in sensory aids for blind and deafblind individuals and compare their sensor and feedback technologies. Secondly, we introduce the proposed system in detail, and highlight its key features and the decisions behind them. We then discuss the experimental procedure for testing this new system, and go on to present the results of several experimental workshops undertaken with two deafblind participants. Finally, we discuss the successes of the current implementation, and opportunities for future work in the field.

## 2. Technology Review

In the UK, there exists a framework of support and mobility assistance for people with MSI, provided by charities such as Deafblind UK [5] and Sense [3]. The most common mobility aid described by these charities is the white cane (white for visual impairment, white and red for audio and visual impairment). There are several types of cane in use worldwide today, preferentially chosen by the user: the symbol cane, guide cane and long cane. The first is only used as a visual marker for awareness to the general public. The latter two are custom made to the height of the user, and are used to sweep the area around the user for upcoming obstacles (guide) and terrain changes (long) [3]. The adaptation and enhancement of canes started with Leslie Kay’s pioneering work in the 1960s with his company Bay Advanced Technologies (BAT), which developed the "K" Sonar-Cane that converts any conventional long cane into a smart cane. [6].

Since then, further products have been designed to enhance the functionality of the white cane, by use of ultrasonic sensors and haptic vibrational feedback. The Ultracane from Sound Foresight Technology [7] uses two sensors (one angled forward with 4m range and the other angled upwards with 1.5m range) which vibrate a pair of buttons with variable frequency to indicate proximity. The technology is embedded seamlessly within a conventional walking cane and the manufacturer reports that it boosts the confidence of the user, unlocking more freedom and allowing faster navigation with its advanced warning of obstacles. However, it is prohibitively expensive for its demographic. In a similar solution, the Miniguide by GDP Research [8] is a hand-held ultrasonic vibration-based detector which can be fitted to a cane, with feedback of five distances up to 8 m fed back via vibrations or beeps in an earpiece. This device is extremely discreet; however, its use requires careful training, the use of a free hand and only acts as a complementary detection system. Moving to wearable solutions, the iGlasses from Ambutech [9] incorporate a pair of ultrasonic sensors and a single vibrational motor—the proximity and surface area control the frequency of the vibration.

There have been numerous devices developed with different monitoring technologies. One such device was implemented by EyeCane [10]. The cane is fitted with a pair of infrared (IR) emitters and sensors to track objects. Information is fed to the user encoded as vibrations through the handle. The authors indicate that the infrared wavelength used offers greater accuracy, and their design expands the detectable field of view, whilst reporting fast training times for users. Similar to the Eyecane, WeWalk [11] uses ultrasound instead of IR pulses for the obstacle monitoring. The main focus of this device is to detect obstacles above waist level, such as sign posts, which are generally not detected by a conventional white cane. The feedback provided is a combination of vibration through the cane and audio information through an ear piece. The resultant product recognizes the need for a comprehensive solution by offering an array of smart features that integrate with the user’s smartphone. An augmented white cane described in [12] has an array of IR LEDs positioned around the cane azimuthally. This device is limited to a fixed scene—the user’s position is triangulated by two IR receivers in the corner of the room and obstacle information is provided by audio messages.

Bai [13] developed a wearable headband with an attached frame for a monitoring system. This system consists of an RGB-D Time-of-Flight (TOF) camera, which is used to perform random sample consensus segmentation on a fixed scene. This allows for the mapping of a traversable area in which the user is safe to walk. The location of obstacles within this area is provided by bone-conducting headphones. The processing of full colour RGB images is a computationally expensive method that requires processing time.

Another system, running solely on a Raspberry Pi 4, was proposed in [14]. Here, the scene is monitored with a simple RGB webcam coupled with an object detection algorithm. Feedback is provided by audio means directly into the ear of the user.

Sound of Vision [15,16] is a 2019 system by Caraiman et al. that detects the scene by means of fusing information from a stereo-vision camera system, an IR-based depth sensor and an Inertial measurement Unit (IMU)—the latter is used to track the orientation of the head/cameras. This then provides environmental reconstruction and segmentation. After modelling a haptic and audio feedback signal for the given scene, it is sent to an earpiece and haptic belt with vibration motors- again, this data fusion approach takes time to model, and the authors report a 7 Hz frame rate using a laptop PC.

A device proposed by Katzschmann [17] consists of two belts connected via Bluetooth: the first is designed to detect and the second is for haptic feedback. The former holds 7 TOF sensors that span 180 deg, worn around the hip—in this configuration the device is not the most discreet solution.. The haptic feedback belt is fitted with five vibratory motors linearly spaced and worn against the chest underneath clothing.

In a change of direction, recent developments in radar technology have given rise to novel, compact and cost-efficient transceivers, opening up the possibility of using radar for scene monitoring. A feasibility study was conducted by Mattia [18], which further supports the use of radar technology for the aid of the multi-sensory impaired. A millimeter-wave (mmWave) radar cane was proposed by Cardillo [19], which combines a mmWave radar with the traditional white cane. The author proposed acoustic or vibrating feedback, and further improved this design by developing a system which distinguishes humans from inanimate objects by detecting vital signs such as breathing/heartbeats [20].

A device that fuses Time-of-Flight, RGB and frequency modulated continuous wave (FMCW) millimeter wave radar sensors to perceive the surrounding obstacles has recently been proposed by Long et al. [21]. The data are processed with various algorithms to obtain more accurate state estimates. For scene feedback, bone conduction headphones connected via Bluetooth are used. This arrangement is not suitable for users with any hearing impairment, due to the instrument-based discretization of feedback sounds.

Lastly, scientists have also explored new regimes for the feedback system. Worthy of note is the device described in [22]. Nguyen et al. successfully demonstrated the Tongue–Placed Electrotactile Display (TED), that provides information about the environment by applying voltages to a matrix of electrodes placed on the human tongue. Another approach that uses air puffs on the forehead to give the direction of a detected object was proposed by [23].

## 3. Proposed System

From the literature, it is clear that there exists the capability to utilise ToF/RGB webcams and radar proximity sensing. The gauge of success in this context, however, is the usefulness of the information obtained for the user. Based on feedback from users, key aspects to consider are the conversion of input stimuli from the environment into an intuitive feedback, and the discretion such a device affords in every day life. The merits of using a cane-mounted system versus a chest, hand or head wearable must ultimately be informed by the intended demographic and field testing.

**Figure 1:**
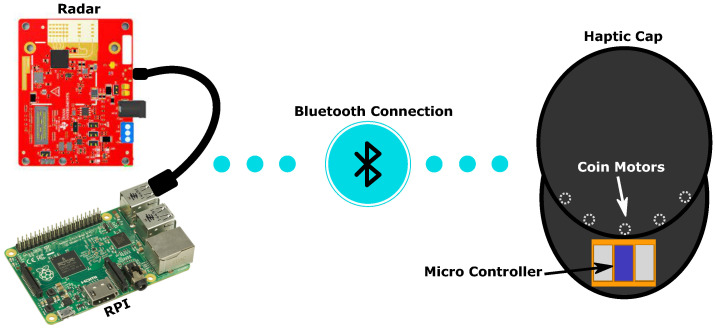
Schematics of the proposed system. The radar transceiver is connected to a Raspberry Pi via USB and sends data about the scene via low energy Bluetooth to the micro-controller on the baseball cap. The micro controller sends pulses to the corresponding coin motors.

Deafblind people can make use of orientation and mobility training to learn the layout of their local environment such as their homes or neighborhood, which can be aided with a white cane or guide dog. However, there remains the threat of obstacles which are dynamic—especially people—which cannot easily be detected by a white cane. Hence, the main objective of the proposed device is to provide a valuable aid enhancement, to inform about dynamic changes in a scene—for instance, people walking within a room, moving objects and obstructions passing by etc., and is intended to complement traditional guiding aids such as the white cane. The proposed device, shown in Figure 1, is the result of discussions and workshops with deafblind participants. For monitoring the scene, we chose an mmWave radar (TI-IWR1642 BOOST). This technology has a number of advantages over the alternative imaging devices (e.g., ToF cameras, RGB cameras) as it is invariant to illumination conditions and hence has the ability to work in both indoor and outdoor environments.

Furthermore, radar waves can also pass through optical scattering such as smoke and fog [24], providing meaningful functionality in these environments. Due to the inherent low imaging capabilities of radar, there are also fewer privacy issues that could arise due to GDPR (General Data Protection Regulation) laws [25]. Lastly, new state of the art radar chips can provide a deeper insight into the dynamics of a monitored scene, since velocity-dependent Doppler radar can provide information on e.g., object motions in dynamic scenes, whilst Micro-Doppler can track vital signs of people and so can distinguish people from objects [20,26].

A key point of the system is the ability to first separate dynamic changes from the static background of a scene. To achieve this, we used the algorithm developed by Huan in [27] that relies on a static clutter removal and clustering steps, which we programmed onto the radar processing chip. This in turn transfers a point cloud of dynamic objects (i.e., people) in the scene to a Raspberry Pi 4 (RPi).The data collected from the radar transceiver are then further processed, before the RPi sends it to the feedback device.

A major factor in the success of a disability aid is in its uptake with the target demographic [28], and so we sought a continuing dialogue throughout the project. Since multi-sensory impaired people also have varying degrees of hearing loss, we implemented an entirely non-acoustic approach for the feedback system. Our discussions with the deafblind participants indicated unfavourable views towards using a belt/sleeve-like device and instead favoured the use of a baseball cap for comfort. Moreover, we consistently noticed that deafblind participants struggled to correctly interpret feedback from a feedback sleeve compared to feedback delivered to the head. The hat is controlled by an Arduino micro-board, which is connected via Bluetooth to the RPi. The vibration feedback is generated by conventional coin motors, akin to that used in mobile phones, which vibrate when a voltage is applied. One can then adjust the intensity and frequency of the vibration by applying different voltages and modulations.

**Figure 2:**
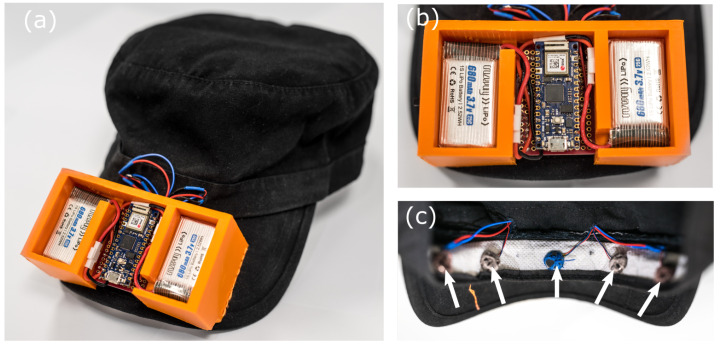
(**a**) Overview of the haptic cap. (**b**) The micro-controller and batteries in a 3D-printed mount attached on top of the visor. (**c**) The exposed coin motors attached in an array to the elastic front panel of the cap.

Our haptic feedback design, shown in Figure 2 uses five motors across the forehead. The angular location of moving people within the scene is then communicated by the location of the coin motors which vibrate accordingly. In the first trials of this project, we also developed a sleeve device intended for the forearm for distance information. However, as commented above, this was poorly received by the deafblind participants on account of comfort and clarity of information. Our solution for conveying depth was to instead encode this information into the intensity of the motor vibrations placed on the forehead.

To limit the complexity of the information transfer, we discretized the intensity encoded distance of detected objects into near (0–2 m) and far (2–6 m) regimes. The baseball cap, shown in Figure 2, could support the micro controller and batteries, but was also flexible enough to isolate the motor vibrations. The sensor design presented above has an angle and distance discretization of 5 × 2 for angle and intensity, to detect objects with a resolution of 10 different locations provided intensity-distance calibration for each user.

The radar is independently capable of detecting up to five people without major issues. However, the limiting factor is finding a suitable method to encode this as haptic feedback to the deafblind participants. Our solution provides only angle and distance information for the closest person in the field of view, since this is likely to be the most important and by increasing the number of moving objects transferred to the user one would also increase the cognitive load on the user drastically.

## 4. Experimental Results

**Figure 3:**
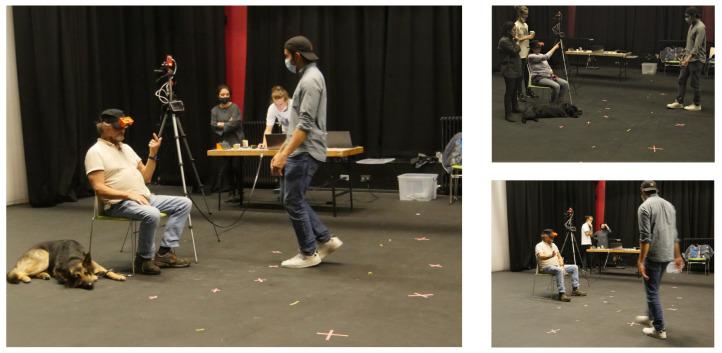
Scenes from the workshops: The deafblind participant sits on a chair adjacent to the radar, whilst the subject to be detected moves through different positions within the field of view of the radar.

To receive relevant feedback about the system, a series of three workshop sessions were scheduled for two volunteers, each with varying degrees of hearing and vision loss, who where invited to participate with the backing of Deafblind Scotland (UK registered charity). In these sessions, the deafblind participants were asked to sit on a chair with the radar placed 40 cm laterally, as can be seen in Figure 3. The field of view of the radar transceiver was mapped out onto the floor, within which the person to be detected was free to move. The workshops consisted of creating different detection regimes to experience and collect constructive feedback on the sensing, comfort and on any problems with the system. Firstly, a single person moved continuously through the scene in a controlled area in front of the participant. Then, two people moved simultaneously in the scene.

At the beginning of the workshops we conducted a short calibration run, to understand how the participants interpret the vibration cues about their surroundings. We realized that both participants required some time to associate the intensity levels to distances. Notably, the coin motor-angle classification was found to be intuitive from the outset. We observed that a deafblind person tends to struggle more with comprehending the feedback and associating both angle and intensity to a single position in space, compared to an individual with no sensory impairment. As an example, when a person is moving just in the near field (same intensity, different motors vibrating) they are easily able to identify the angle. In contrast, when the subject to be detected moved freely between the near and far field, the discretisation of depth and angle proved more challenging for the participant to accurately classify.

**Figure 4:**
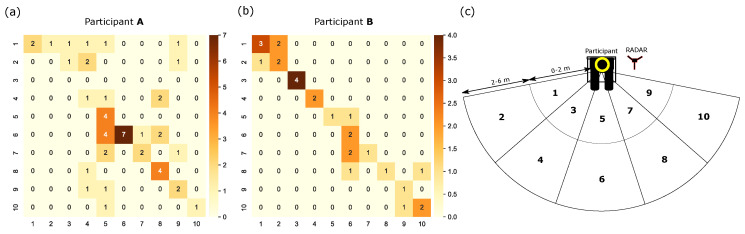
(**a**,**b**) The confusion matrices of a single person moving in the scene through locations 1–10 for deafblind participant A and B. (**c**) A schematic of our experimental setup during the workshop with locations 1–10 indicated.

Both participants also took part in quantitative testing, the results for which can be seen in the confusion matrices in Figure 4a,b. During each test, the target person moved within the radar field of view, stopping within each numbered segment at random, shown in Figure 4c. This allowed the participants to make a series of predictions of the target location, and the predictions and the true target locations were recorded. Participant A was found to predict accurately (when prediction segment and true target segment match) 47% of the time; however, including predictions that fell within a neighboring segment, this brings the accuracy to 80%. Participant B performed better, with 68% of predictions correctly identifying the target segment and 100% of predictions at least within a neighboring segment. Note that we classify neighboring segments as those sharing a border line (e.g., segments 2 and 3 are neighbors to 1). This allows us to evaluate the results beyond a simple binary classification.

## 5. Conclusions

From the proof of concept results shown, we believe that mmWave sensing coupled to haptic feedback holds promise for future applications of portable sensing technology for improving the lives of people with multi-sensory impairment. These applications may include real-world scenarios such as portable detection of moving people, bicycles and cars, detecting when someone enters a room, and detection and classification of inanimate objects or animals. The feedback from the two deafblind participants proved indispensable in the development of the various iterations of the haptic cap. Due to their past experience with other projects within Deafblind Scotland they were able to use their knowledge to describe their needs in a very valuable way. Much of the workshop time was spent calibrating and adjusting settings to gauge the preliminary reactions to the technology. Initially, there were a total of six participants planned for this project, but due to COVID and other restrictions, we had to reduce the number of participants to only two for the workshops. In the future, we would like to undertake a more comprehensive and wide ranging set of field tests, involving a significant number of participants with varying degrees of multi-sensor impairment.

We note that in contrast to some studies in the literature using non-disabled participants, there is an inherent issue with calibration for deafblind users. This is due to not being able to associate and subsequently memorise subject locations with visual prompts. Instead, our calibration involved dictating to the participant the subject location as they moved through the field of view, in the near and far distances, and compared near and far field locations for specific angular locations.

One issue which arose during these workshops was the varying degrees of hearing loss and asymmetrical hearing of the participants, which results in different sensory experiences for the users. The haptic vibrations are sensed by both the nerve endings in the skin and the frontal bone of the forehead—as such, there is an unknown interplay between these sensations which is exposed to the physiology of the individual. An asymmetry or deviation from an expected sensory response in the forehead and ears may affect the distance location of the perceived signal. We believe this to be a factor in the discrepancies between Participant A and B, where A disclosed different levels of hearing between their ears. In future tests, we can implement individual intensity tuning of the haptic feedback motors, such that the participants could then perceive the vibrations to their own suitability. This demonstrates that such a sensory device might need to be calibrated to each user to prevent discomfort and inaccurate interpretation of the vibrations. Additionally, one could feasibly add further mmWave sensors to monitor 360 degrees around the user or increase the number of people to be detected. However this would inevitably complicate the feedback to the user—care would need to be taken to avoid exceeding the acceptable cognitive load.

Overall, the performances in the static classification test of the 10 different positions show a positive outlook for this technology. We have demonstrated how this approach may be developed both in hardware and algorithm with a great deal of potential for further study and testing.

## Data Availability

Data used to produce the figures shown in this paper are available in https://dx.doi.org/10.5525/gla.researchdata.1323 (Last accessed on 4 September 2022) by request only.
